# The spatial distribution of salt tolerant bacteria and other soil parameters under different agricultural systems of the Indian Sundarbans

**DOI:** 10.1371/journal.pone.0333742

**Published:** 2025-10-08

**Authors:** Sreemoyee Mitra, Priyanka Das, Ritabrata Karmakar, Pabitra Banik, Arup Bose

**Affiliations:** 1 Agricultural and Ecological Research Unit, Biological Science Division, Indian Statistical Institute, Kolkata, West Bengal, India; 2 Applied Statistics Unit, Indian Statistical Institute, Kolkata, West Bengal, India; 3 Statistics and Mathematics Unit, Indian Statistical Institute, Kolkata, West Bengal, India; Remote Sensing Application Centre, INDIA

## Abstract

Salinization is a major contributor to soil degradation and poses a substantial threat to agriculture. In India, approximately 6.73 million hectares of lands are affected by salinity, with the Indian Sundarbans playing a critical role in coastal salinity issues. Our investigation focuses on the agricultural soil in the Indian Sundarbans, shifting away from the previously studied mangrove soil, and delving into its chemical and microbiological characteristics. We aimed to find the spatial variation in soil salinity, nitrogen level, soil organic content, and salt-tolerant bacteria along with any possible effect of bacterial population on soil chemical attributes. This was achieved through soil lab analysis, geo-statistics and analysis of parameters by ArcGIS. Notably, the central Sundarbans exhibits higher salinity levels compared to its eastern and western regions. Salt-tolerant bacteria are more abundant in specific areas, including villages of *Bakkhali, Sibrampur, Patharpratima*, and certain villages of *Sagar Island.* While the presence of salt-tolerant bacteria in saline regions is influenced by the types of crops cultivated, most soil characteristics tend to vary primarily due to spatial factors rather than cropping patterns. Further research should focus on the beneficial effects of salt-tolerant bacteria on the Available Nitrogen content in the soil. These findings will aid in understanding microbial growth in saline conditions in the future and benefit crop growth in such challenging environments.

## 1. Introduction

Salinization is a significant factor in soil deterioration, and is a serious threat to agriculture. Soil salinity is commonly measured by Electrical Conductivity (EC) of soil saturation extract (in terms of dS/m), and any soil with EC > 4dS/m is considered saline [1] Recent reports from the Food and Agriculture Organization [2] of the United Nations indicate that approximately 1.4 million hectares (ha) of global soil is saline. This includes around 10% of irrigated cropland, and 10% of rain-fed cropland. In India, approximately 6.73 million ha is salt affected, consisting of 2.96 million ha of saline soil, and 3.77 million ha of sodic soil (soil with an excess of sodium ions) [3]. Nearly 75% of salt-affected soil in India is in the states of Gujarat (2.23 million ha), Uttar Pradesh (1.37 million ha), Maharashtra (0.61 million ha), West Bengal (0.44 million ha), and Rajasthan (0.38 million ha) [4]. Salinity is primarily found in coastal areas, with the Indian Sundarbans, located in West Bengal, being a major contributor to coastal saline soil [5,3]. The Sundarbans is a vast deltaic complex with mangrove forests extending from India to Bangladesh, and it is a key biodiversity hotspot. This dynamic ecosystem is constantly influenced by both environmental and anthropogenic factors [6].

The mangrove habitat is characterized by its saline environment and unlike conventional agricultural lands, saline agricultural areas face unique challenges. These include limited water availability for crops, essential nutrient deficiencies—particularly low levels of phosphorus and potassium—deteriorated soil structure, and heightened vulnerability to soil erosion. All these factors lead to low crop productivity and therefore, have a negative impact on the economic conditions of the local farmers. The Indian Sundarbans also suffers from frequent tropical cyclones, traditional agricultural practices and unpredictable weather patterns [7,8]. Globally, agriculture follows different patterns of mono-cropping, mixed cropping and crop rotation (seasonal or yearly), shifting cultivation or intercropping etc. [9], and farming methods in Indian Sundarbans is no different. To combat the saline environment, farmers resort to different practices like raised bed sowing, paddy straw mulching, deep ploughing, and use of excessive chemical fertilizers and pesticides [10]. Differences in land use pattern in the Indian Sundarbans create a unique micro biota [11]. Additionally, with the change in salinity and detritus load, the microbial life of this area varies. For instance, in the freshwater region with low salinity, there are more mesophiles and chemoautotrophs than in areas with a high salt content. *Halobacterium* spp., *Halococcus* spp. and *Vibrio* spp. dominate the region [12]. One challenge of working in the Indian Sundarbans is the adverse weather conditions and inaccessibility of the land.

Previously it has been seen that, microbial abundance varied significantly between untreated soils, soils treated with chemical fertilizers and those treated with manure. [13]. This compels the study to understand how changes in agricultural method effect the bacterial communities. Although microbial habitats of the Indian Sundarbans have been extensively studied, research has mostly concentrated on the mangrove regions. Due to this, the halophiles and halotolerant organisms in the agricultural regions have not been well explored. Previously it has been seen that halotolerant bacteria helps in saline soil management in saline agricultural areas [14], and therefore it would be very interesting to see how changes in agricultural method have an impact on them and vice-versa. Addressing existing research gaps, we formulated the objectives of the work. This included analyzing the variation and patterns of soil characteristics across agricultural lands (included two cropping systems, mono-cropping and mixed cropping) and investigating how soil parameters—regardless of cropping system—affect the growth and presence of salt-loving bacteria. The goal was to understand the relationship between soil microbial and chemical parameters, and to determine whether these factors change with different agricultural patterns or are influenced more significantly by climate, topography, use of fertilizers, and irrigation practices.

## 2. Materials and methods

### 2.1 Study area and collection of soil samples

**Fig 1:**
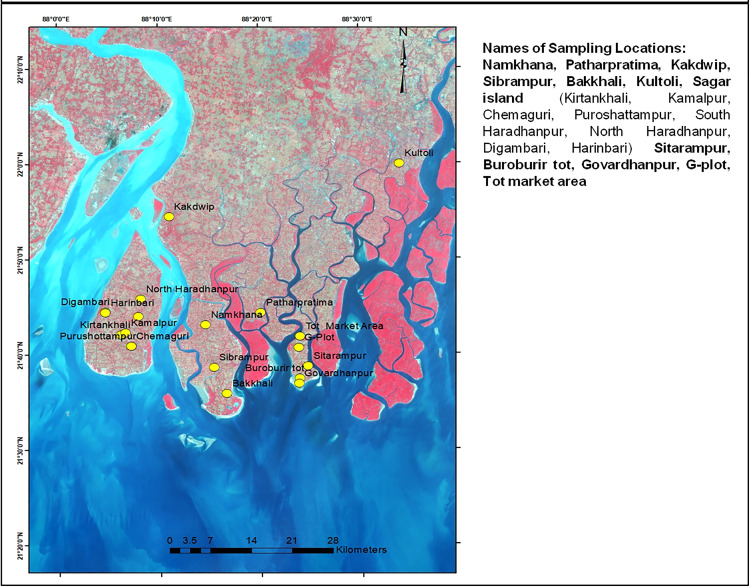
The 19 sampling locations in Indian Sundarbans.

Soil samples were collected from the agricultural regions of the Indian Sundarbans having soil type as Typic Halaquepts of Inceptisols, following the USDA soil taxonomy. As shown in Fig 1, 80 soil samples were collected from total 19 sites belonging to 5 Blocks— *Namkhana* (villages- *Namkhana, Sibrampur, Bakkhali*), *Kakdwip, Patharpratima* (villages- *Patharpratima, Sitarampur, Buroburir tot, Govardhanpur, G-plot,* and *Tot market area*) *Kultoli* and *Sagar Island* (*Kirtankhali, Kamalpur, Chemaguri, Purushottampur, North* and *South Haradhanpur, Digambari and Harinbari*). Location co-ordinates and type of agriculture system is listed in Table 1 and S1 File. The 5 blocks were selected on the basis of their accessibility and geographical distances, intentionally to represent a larger area of the Indian Sundarbans. The sampling area covered an area of approximately 1200 sq. km. The sampling locations were selected according to the convenience of access and the purpose of acquiring both mixed and single crop type of soils. Soil samples were aseptically collected in triplicates at a depth of 12–15 cm from agricultural fields after crop harvest, and kept in ziplock bags at room temperature until further use. Post-harvest soil samples were sampled since they offer insights into the nutritional requirements of the soil, facilitating better crop yields in the following season [15].

**Table 1:** Result of soil analysis performed on the 80 collected soil samples.

	Sl. No.	Location Name	pH	Electrical Conductivity(dS/m)	Bacterial Count (*10^6)	20% NaCl tolerating bacteria	10% NaCl tolerating bacteria	Organic Carbon(%)	Available Nitrogen(kg/ha)	Microbial Biomass C (µg/g)
MONOCROPPING	S1	Namkhana	5.42	5.31	7.6	3	10	1.8	10.45	781.15
	S2	Patharpratima	6.64	4.05	5.2	3	11	2.09	16.73	492.86
	S3		7.26	4.09	3	1	5	2.92	35.54	390.76
	S4		6.88	6.26	3.3	1	6	0.94	10.45	150.52
	S5	Kultoli	7.13	4.86	6	1	2	1.27	54.36	510.88
	S6		7.84	4.17	3.6	0	1	1.51	13.59	480.85
	S7		7.2	5.03	4.6	1	3	1.08	7.32	340.91
	S8		7.47	5.55	3.2	2	5	1.02	10.45	241.21
	S9		7.11	3.82	5	1	3	2.65	23	661.03
	S10		6.99	4.49	1.06	2	4	1.91	15.68	600.97
	S11	Kirtankhali	5.39	2.25	3.9	0	1	2.73	18.82	1051.42
	S12		6.64	3.52	4.1	2	0	1.89	12.54	811.18
	S13		7	4.21	3.9	1	0	1.28	16.73	510.88
	S14	Kamalpur	7.27	2.37	3.1	0	2	2.43	12.54	721.09
	S15		7.1	2.73	2.5	0	1	2.57	12.54	811.18
	S16		6.98	2.85	3.7	0	4	2.69	29.27	841.21
	S17	Chemaguri	7.43	3.95	4.8	2	5	2.06	32.41	841.21
	S18		7	3.84	2.3	1	4	0.87	12.54	1171.54
	S19	Purushottampur	7.28	4.17	4.7	0	6	1.66	18.82	1231.6
	S20		7.46	4.21	4.5	1	1	0.83	23	460.83
	S21	South Haradhanpur	7.39	4.47	3.3	1	3	1.62	25.09	600.97
	S22		6.2	4.01	2.5	0	3	2.07	24.04	661.03
	S23		5.73	2.65	3.4	2	4	2.32	13.59	691.06
	S24	N. Haradhanpur	6.88	0.59	4.2	0	2	2.24	14.63	1111.48
	S25		7.38	0.45	3.3	0	1	1.99	19.86	480.85
	S26		7.21	1.52	3.8	1	2	1.35	24.04	552.92
	S27	Digambari	7.24	2.53	5.4	0	1	2.18	14.63	600.97
	S28		7	1.8	2.3	2	3	2.11	13.59	416.79
	S29	Harinbari	6.42	0.87	3.1	1	0	2.49	10.45	1021.39
	S30		6.27	4.26	3.5	0	5	1.43	9.41	1201.57
	S31	Sitarampur	6.65	0.54	2.9	2	6	1.08	23	1231.6
	S32		6.42	1.37	9.2	0	6	1.4	25.09	1111.48
	S33		7	0.51	7.1	0	4	2.67	23	1051.42
	S34	Govardhanpur	5.75	0.25	8.7	0	3	1.91	30.31	1141.51
	S35		7.47	2.34	3.1	1	4	1.46	24.04	210.58
	S36		6.68	1.24	2.5	0	1	1.92	33.45	300.67
	S37	G-Plot	6.54	1.24	2.7	0	2	2.23	29.27	330.7
	S38		6.88	1.99	8.1	0	3	0.43	23	420.79
	S39		7.09	1.53	6.7	0	3	1.81	26.13	510.88
	S40	Tot Market Area	5.92	0.3	6.4	0	1	1.69	27.18	360.73
MIXEDCROPPING	S41	Namkhana	7.98	3.85	6.7	1	8	2.45	7.32	590.76
	S42	Patharpratima	6.18	4.08	3.4	0	10	1.87	19.86	240.61
	S43	Kakdwip	7.92	3.11	2.4	2	12	2.14	9.41	320.49
	S44		7.53	3.21	6.6	0	10	2.27	16.73	540.91
	S45	Sibrampur	6.12	4.08	4	1	3	2.15	11.5	120.49
	S46		5.7	3.14	5	0	3	2.55	15.68	841.21
	S47		5.22	6.19	7.9	1	9	1.7	24.04	180.55
	S48	Bakkhali	7.3	7.46	6.3	2	10	0.98	23	240.61
	S49		5.47	6.75	10.9	1	13	1.44	45.99	180.55
	S50		5.67	5.44	6.6	2	7	1.75	16.73	570.94
	S51		5.32	4.23	8.6	4	7	1.69	50.18	480.85
	S52	Kirtankhali	7.6	4.65	5	1	0	1.18	13.59	781.15
	S53		7.41	4.83	6.9	2	4	0.99	27.18	811.18
	S54		7.61	4.75	7.8	0	2	1.05	25.09	540.91
	S55		7.5	3.95	9.5	0	3	1.76	15.68	1021.39
	S56		7.43	4.01	11.1	1	2	1.78	15.68	480.85
	S57		7.43	3.47	8.7	3	6	1.66	27.18	1021.39
	S58		7.5	5.76	3.4	1	5	1.22	18.82	1171.54
	S59		7.45	3.26	5	1	3	2.88	23	751.12
	S60	North Haradhanpur	7.35	3.24	7.7	2	3	2.58	16.73	721.09
	S61		7.37	2.57	6.2	0	2	1.36	30.31	961.33
	S62		7.48	2.27	2.2	0	1	1.24	23	901.27
	S63		7.47	3.35	4	0	2	1.5	9.41	1171.54
	S64		7.22	2.8	8.6	0	3	1.56	13.59	1111.48
	S65		7.49	1.31	5.1	0	4	2.32	18.82	1051.42
	S66	Harinbari	6.66	0.53	5.6	0	5	1.45	7.32	1081.45
	S67		7.31	0.52	7.9	2	4	1.57	13.59	931.3
	S68		7.15	3.19	11.8	2	4	2.12	21.95	1111.48
	S69		6.85	3.41	5.2	1	7	1.54	18.82	1171.54
	S70	Sitarampur	7.36	0.92	6.4	2	13	1.45	24.04	1411.78
	S71		7.44	0.21	10.7	3	7	1.65	33.45	1201.57
	S72		7.58	0.26	12.8	0	6	2.08	24.04	691.06
	S73		7.53	0.3	7.7	0	9	1.87	28.22	661.03
	S74	Buroburir tot	7.98	0.5	4.2	0	3	0.68	34.5	300.67
	S75		7.93	0.37	3.7	0	1	0.72	28.22	360.73
	S76		7.81	2.56	2.6	0	5	0.89	23	480.85
	S77	Tot Market Area	8.19	0.2	2.4	0	5	0.4	31.36	670.64
	S78		5.47	0.23	2.8	0	4	1.14	31.36	570.94
	S79		5.52	0.17	2.9	0	4	0.52	26.13	780.55
	S80		4.98	0.82	3.3	0	7	1.28	19.86	661.03

Since the soil samples were collected from private agricultural lands and not from the forested areas of the Indian Sundarbans, there was no necessity of acquiring additional permission.

The samples were collected using *Stratified Purposive Random Sampling,* in autumn of 2020, and in both autumn and spring of 2021. The monocropping systems consisted of only paddy cultivation whereas the mixed crops consisted of paddy along with vegetables. The source of water for the crops was primarily rain and irrigation from nearby ponds. All the sampled locations had similar patterns for fertilizers, mostly relying on urea, DAP, and superphosphate. The farmers also applied cow dung or vermicompost (rarely), as given in detail in S1 File (S1 Table 1).

### 2.2 Soil microbial and physico-chemical parameters

Microbial analysis of the samples was done with the wet soil. For the chemical analyses, the soil samples were air-dried and finely ground (<0.20 mm) before conducting experiments.

#### 2.2.1 Microbial analysis.

- Isolation of bacteria: For bacterial isolation, we followed the protocols of serial dilution and spreading of inoculum from Cappuccino & Sherman [16], with slight modifications. One gram of each soil sample was dissolved in 9 ml of 0.9% saline. 100 μL samples from successive serial dilutions (10^−3^, 10^−5^, and 10^−7^) were spread on Nutrient agar. These agar plates were enriched with NaCl at 2% (0.34 M), 5% (0.85 M), and 10% (1.71 M) and kept at 37 ± 2°C till the appearance of bacterial growth, for approximately 48h-72h. The entire experiment was set with the precise aim of facilitating the growth of only salt-tolerant bacteria.
- Halotolerance assay of isolates: Bacterial isolates grown in the salt-supplemented plates were further streaked for pure culture. For checking the intensity of salt tolerance of the individual isolates, each of them was inoculated on agar plates supplemented with two concentrations of NaCl of 10% (1.71 M) and 20% (3.42 M). Inoculation was done with a uniform concentration of inoculum (OD600 = 0.5) from all the isolates, and incubated at 37 ± 2°C for 7 days, so as to induce maximum time for growth.

#### 2.2.2 Estimation of the microbial biomass carbon in the soil samples.

Soil respiration expresses the gas exchange of aerobic and anaerobic metabolisms. Hence it is one of the soil parameters that is measured for understanding the microbial activity in the soil. The addition of a substrate helps in the process, as it aids in the decomposition of the already present organic mass in the soil. We followed this substrate induced respiration (SIR) method using the protocol from Alef [17] and FAO [18] with necessary modifications. Ten grams of soil sample (using a 2 mm sieve) was taken in a 500 ml conical flask and moistened with 1 ml of 0.5% glucose solution. 5 ml of 0.1(M) of NaOH was hung inside the conical flask and was closed with an airtight cork. The setup was kept for 5 hours at 25°C, and then the collected CO_2_ in the NaOH solution was titrated with 0.05(N) HCl, using Phenolphthalein as an indicator. The Microbial biomass carbon in the soil was then found using the formula mentioned in Ananyeva et al., [19]:


$$MB_{SIR}=\text{SIR}\times \text{40}.\text{04}+\text{0}.\text{37}.$$

#### 2.2.3 Physio-chemical parameters of soil.

The following parameters were measured using sieved dry soil with the methods given in Singh et al., [20], mutatis mutandis.

- *Soil pH*: This was measured using a pH meter [Make: EUTECH INSTRUMENTS, part of Thermo Fisher Scientific, Made in Singapore] using soil:water ratio of 1:2.5.
- *Soil salinity* (expressed in dS/m): It was determined by measuring the electrical conductivity (EC). The soil samples were air-dried and finely ground (<0.20 mm). EC was measured using a Conductivity Meter [Make: LABARD, LIM-193] in the units of dS/m, with a soil:water ratio of 1:5.
- *Soil Organic Carbon* (expressed in %): This was measured by the Walkey-Black method using Potassium dichromate and Mohr’s salt.
- *Available Nitrogen* (expressed as kg/ha): This essential factor for plant take-up was estimated using a Kjeldahl distillation unit [Make: BOROSIL] with Potassium permanganate and Boric acid [as indicator].

### 2.3 Spatial distribution of salt loving bacteria, EC and other soil factors

**Fig 2:**
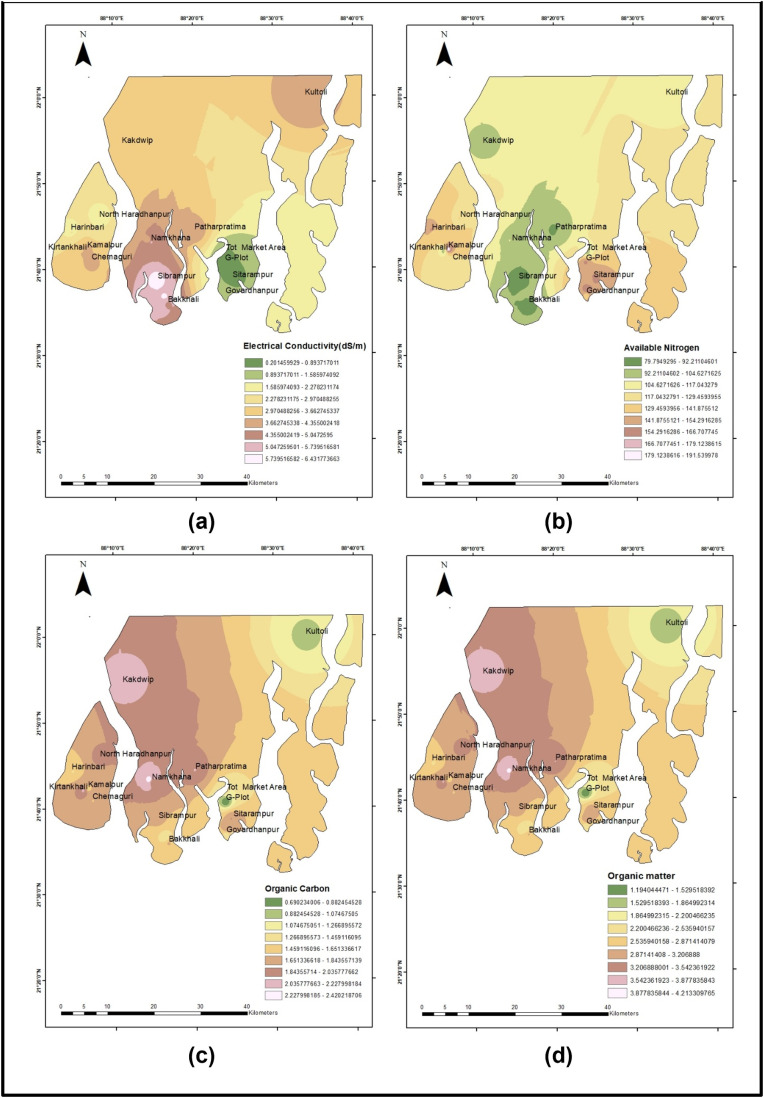
Spatial analysis of soil for: (a) Electrical Conductivity (dS/m), (b) Available Nitrogen (kg/ha), (c) Organic Carbon (%), and (d) Organic Matter (%).

**Fig 3:**
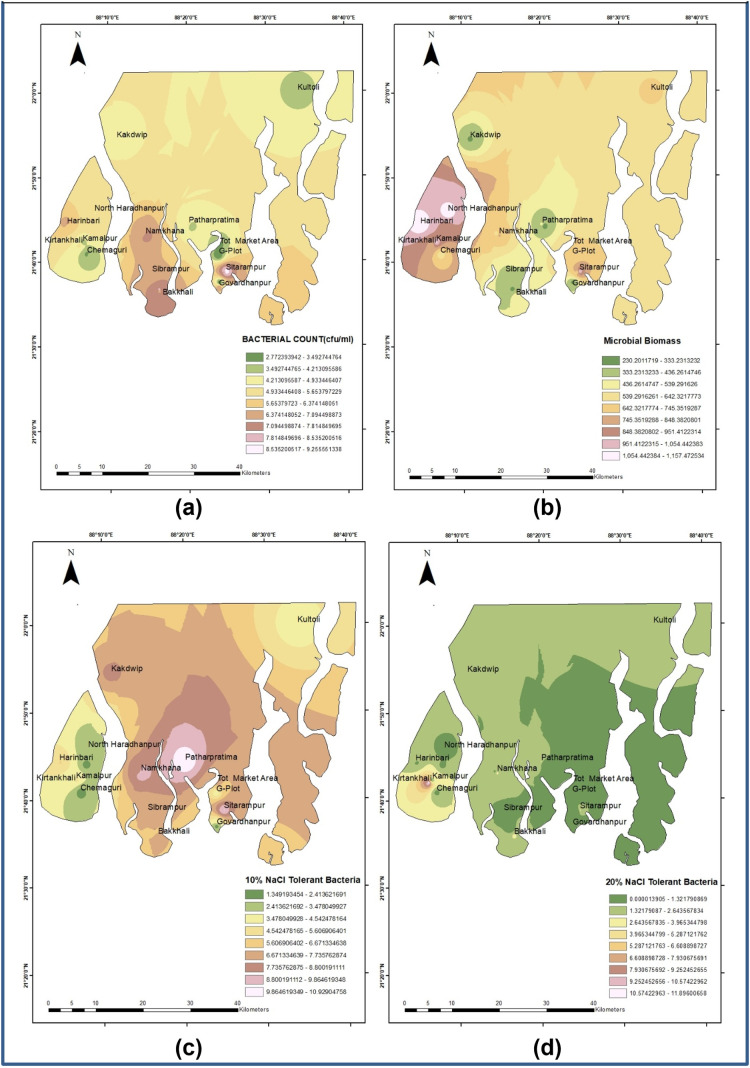
Spatial analysis of soil for: (a) Bacterial Count (cfu/ml), (b) Microbial Biomass Carbon, (c) 10% NaCl tolerating bacteria, and (d) 20% NaCl tolerating bacteria.

The sampling site map was prepared using Landsat 9, acquired in December 2022 under cloud-free conditions. In Figs 2 and 3, with the help of ArcGIS 10.8, we show the spatial pattern of (a) bacterial growth, (b) number of bacterial species, and (c) the electrical conductivity.

The spatial spread of the different soil parameters was mapped similarly. Multiple samples were taken from each site under various conditions/factors. Thus, each layer of the map is prepared for a single factor. For a lucid exhibition of spatial variations, some sites were magnified for each condition/factor.

### 2.4 Statistical analysis

All statistical analyses were conducted using R-Studio, under the standard assumption of statistical independence of the observations.

First, box, bar, and quantile–quantile (Q–Q) plots were used for an exploratory analysis. *Box plots* display the median, interquartile range (IQR), and outliers, with the box representing the middle 50% of the data and the whiskers extending to the smallest and largest values within 1.5 × IQR (inter-quartile range). *Bar plots* show the relative frequency of count values. These plots facilitate visual comparison of data sets. *Q-Q plots* assess normality by plotting sample quantiles on the y-axis against standard normal quantiles on the x-axis. An approximately linear pattern indicates normality.

Next, we compared mono and mixed cropping systems for each soil characteristic, except for the counts of 10% and 20% NaCl-tolerating bacteria, using Welch’s t-test and the Wilcoxon Mann–Whitney U test. *Welch’s t-test* assesses equality of means for two populations with possibly unequal variances and sample sizes, under the assumption of normality. In case of non-normality, we applied the *Wilcoxon-Mann-Whitney U test.* Then, the counts of 10% and 20% NaCl-tolerating bacteria were compared for mono and mixed cropping systems using generalized linear models (GLMs) with bacterial count as the response variable and the type of cropping system as the explanatory factor. These two bacterial counts were analyzed separately, as they represent count data with a small number of distinct values. Subsequently, a deviance-based chi-square test was performed to assess the significance of the dependence of bacterial counts on the type of cropping system. This test compares the log-likelihoods of the proposed model and a null model in which the response is assumed to be independent of the predictor. For each of these characteristics, the analysis was conducted twice using two different link functions for the GLM: Poisson and negative binomial. Finally, we assessed the dependence of bacterial count on the other characteristics using ordinary linear regression, with bacterial count as the response variable and the type of cropping system, electrical conductance, organic carbon, available nitrogen, and microbial biomass carbon as explanatory variables. Since bacterial count is measured in units of 10⁶, it was treated as a continuous variable, making linear regression appropriate. To evaluate model fit, we examined the plots of residuals (differences between observed and predicted values) versus the fitted values. We also used the *Akaike Information Criterion* (AIC) to compare and choose between different models.

## 3. Results

### 3.1. Chemical and microbial analysis of soil

Table 1 gives the result on soil analysis. The data represented here are the mean of the values provided in S1 Table 2 in S1 File.

### 3.2. Spatial analysis of the soil parameters

We utilized ArcGIS to create maps illustrating the spatial patterns of soil parameters. These maps (see Figs 2 and 3) demonstrate how soil characteristics vary across the sampling areas.

**Fig 4:**
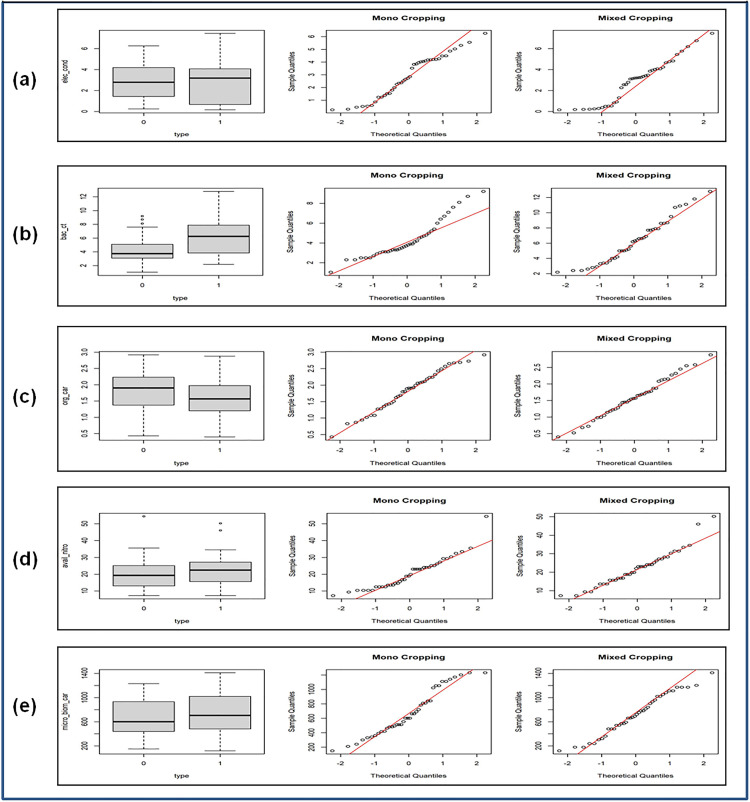
Box and Q-Q plots for (Mono and Mixed Cropping). (a) Electrical Conductivity, (b) Bacterial Count, (c) Organic Carbon, (d) Available Nitrogen, (e) Microbial Biomass Carbon.

Fig 2 shows the pattern of the soil properties—Electrical Conductivity (EC), Available Nitrogen, Organic carbon, and organic matter across the areas. EC is higher in areas of *Sibrampur* and *Bakkhali* and lowest near *G-plot* with a range of 0.2 dS/m to more than 6 dS/m across the sampled locations,indicating a variation in salinity across the lands. The images shown in Fig 2 and the subsequent statistical analysis (Fig 4a and Table 6) revealed that the differences in EC were not attributable to variations in agricultural practices. In contrast, the plant Available Nitrogen is seen to be higher in the areas of Sagar islands, islands of *Sitarampur*, and *G-plot* area, while it is seen to be comparatively lower in the areas of *Kakdwip, Sibrampur, Patharpratima*, and *Bakkhali*, with a range of as high as 190 kg/ha to as low as 79 kg/ha across the regions. Organic Carbon and Organic Matter both were seen to be in the higher range in areas of *Kakdwip* and *Namkhana* whereas, it was lower in *Kultoli* and *G-plot*. These patterns can be seen clearly in Fig 2.

**Table 6:** p-values for comparing mono-cropping and mixed-cropping systems across various factors.

Factor	p-value	
	Welch’s t-test	Mann-Whitney U testU UU
Electrical Conductivity	0.9885	0.6930
Bacterial Count	0.000629	0.001671
Organic Carbon	0.08907	0.08904
Available Nitrogen	0.4449	0.3838
Microbial Biomass Carbon	0.5429	0.5574
	Poisson Regression Model	Negative Binomial Regression Model
20% NaCl tolerating Bacteria	0.7139	0.7367
10% NaCl tolerating Bacteria	0.00000451	0.0008086

Similar to what is shown in Fig 2, Fig 3 shows the nature of soil properties— Bacterial Count (cfu/ml), Microbial Biomass Carbon, 10% NaCl tolerating bacteria, and 20% NaCl tolerating bacteria across the areas. Bacteria tolerant to salt are primarily found in *Sitarampur* and *Bakkhali*, while their presence is low in *Chemaguri, G-plot,* and *Kultoli*. In contrast to the pattern of the presence of salt-tolerable bacterial populations, the microbial biomass carbon is present at a higher level in areas of *North Haradhanpur, Digambari*, and *Harinbari* (>1100) and at a lower level in *Kakdwip, Patharpratima,* and *Govardhanpur* (<200). Both 10% NaCl-tolerating bacteria, and 20% NaCl-tolerating bacteria is seen to be present mostly in areas of *Patharpratima, Sibrampur,* and *Kakdwip*, while their presence is low in areas of Sagar islands.

In Fig 3, it is evident that the counts of salt-tolerant bacteria, specifically those tolerant to 10% NaCl, as well as microbial biomass carbon, are higher in areas with mixed cropping. The patterns of salt-tolerant bacterial counts and microbial biomass carbon appear similar across the sampling locations. Further analysis indicates a significant difference between the monocropping and mixed cropping groups concerning bacterial counts and the number of bacteria tolerant to 20% NaCl.

From the datasets and figures, we conclude the presence of more salt-tolerant bacterial growth in areas of *Patharpratima, Sibrampur, Bakkhali,* some villages of *Sagar Island*, and *Sitarampur*. However, it may be noted that this has no relation to the microbial biomass carbon, as the latter was measured based on the total microbial population that includes both salt tolerant and non-halotolerant microbes.

### 3.3 Statistical analysis of the soil parameters

The statistical analysis focused on two main issues: (1) the effect of the type of cropping system on soil parameters and (2) the dependence of bacterial count on other factors. The results are presented in the following subsections.

#### 3.3.1 Comparison of different soil parameters: Mono vs Mixed Cropping system of cultivation.

**Fig 5:**
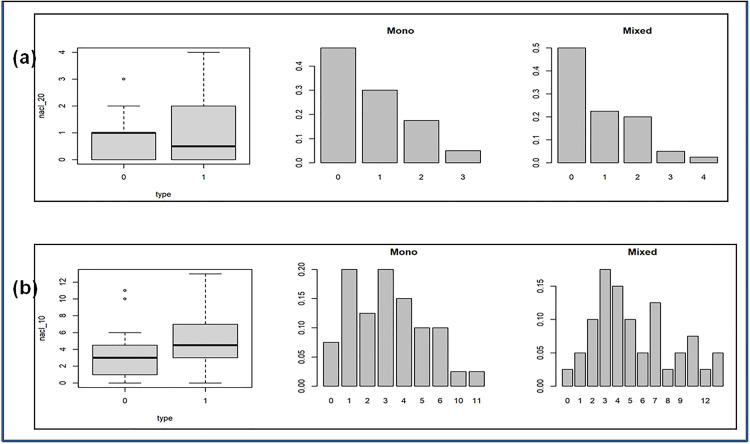
Box plot and relative frequency bar plot. (a) 20% NaCl tolerating Bacteria and (b) 10% NaCl tolerating Bacteria for the two cropping systems.

The distributional pattern of different soil characteristics and the comparison between mono and mixed-cropping systems were done using box plots, Q-Q plots, and the bar plots. The box plots suggest differences in the distribution across the two cropping systems. The Q-Q plots show significant deviation from normality, and hence we also applied the Wilcoxon-Mann-Whitney U test in addition to Welch’s t-test to compare the samples. The relevant plots are shown in Figs 4 and 5, and the results of the tests are presented in Table 6.

The bar plots in Fig 5 suggest a Poisson or negative binomial link function for a GLM. For the negative binomial model, an additional parameter, θ, determines the shape of the distribution. The regression and deviance analyses are summarized in Tables 2–5.

**Table 2:** Regression analysis of number of 20% NaCl tolerating bacteria in soil samples.

Poisson Regression – nacl 20 ~ type				
	Estimate	Std. Error	z value	Pr(>|z|)
(Intercept)	−0.2231	0.1768	−1.26	0.2068
Type	0.0896	0.2446	0.37	0.7141
Negative Binomial Regression – nacl 20 ~ type				
	Estimate	Std. Error	z value	Pr(>|z|)
(Intercept)	−0.2231	0.1921	−1.16	0.2453
Type	0.0896	0.2667	0.34	0.7368
Shape (θ)	4.4311	5.27		

**Table 3:** Analysis of deviance for number of 20% NaCl tolerating bacteria in soil samples.

Poisson Regression – nacl 20 ~ type					
	Deviance (dev)	Deg. of freedom (df)	dev.difference	df.difference	p-value (P(>dev.diff))
Null Model	102.8086	79	–	–	–
Model with “Type”	102.6743	78	0.1343	1	0.7139
Negative Binomial Regression – nacl 20 ~ type					
	Deviance (dev)	Deg. of freedom (df)	dev.difference	df.difference	p-value (P(>dev.diff))
Null Model	88.8729	79			
Model with “Type”	88.7599	78	0.1130	1	0.7367

**Table 4:** Regression analysis of number of 10% NaCl tolerating bacteria.

Poisson Regression – nacl 10 ~~ type				
	Estimate	Std. Error	z value	Pr(>|z|)
(Intercept)	1.1863	0.0874	13.58	≈ 0.0000
Type	0.5001	0.1107	4.52	≈ 0.0000
Negative Binomial Regression – nacl 10 ~ type				
	Estimate	Std. Error	z value	Pr(>|z|)
(Intercept)	1.1863	0.1129	10.51	≈ 0.0000
Type	0.5001	0.1500	3.33	≈ 0.0009
Shape (θ)	4.886	1.72		

**Table 5:** Analysis of deviance for number of 10% NaCl tolerating bacteria in soil samples.

Poisson Regression – nacl 10 ~ type					
	Deviance (dev)	Deg. of freedom (df)	dev.difference	df.difference	p-value (P(>dev.diff))
Null Model	174.93	79	–	–	–
Model with “Type”	153.8952	78	21.0348	1	0.00000451
Negative Binomial Regression – nacl 10 ~ type					
	Deviance (dev)	Deg. of freedom (df)	dev.difference	df.difference	p-value (P(>dev.diff))
Null Model	97.6946	79			
Model with “Type”	86.4732	78	11.2214	1	0.0008086

To compare the two samples corresponding to mono and mixed cropping systems, we performed both Welch’s t-test and the non-parametric Mann-Whitney test. Table 6 presents the results of Welch’s t- and Mann–Whitney tests for all soil characteristics except for the counts of 10% and 20% NaCl-tolerating bacteria. These show that among the characteristics analyzed, only the bacterial count differs significantly between cropping systems at the 5% level (p < 0.05).

For the 10% and 20% NaCl-tolerating bacterial counts, generalized linear models (GLMs) with Poisson and negative binomial link functions were employed. The results of the GLM fit are presented in Tables 2 and 4, respectively. The deviance analyses for the chi-square tests of these factors are provided in Tables 3 and 5. The “null model” represents a model without any explanatory factors. The model incorporating “type” (“type 0” and “type 1” for the mono and the mixed-cropped systems respectively) as an explanatory factor is compared against the null model.

Both the GLMs led to the same conclusions that, at the 5% significance level, the count of 10% NaCl-tolerating bacteria depends on the type of cropping system, whereas the count of 20% NaCl-tolerating bacteria does not.

The “p” values for all the tests are given in Table 6. In summary, significant differences between the two cropping systems were observed only for the bacterial count and the number of 10% NaCl-tolerating bacteria. All other factors showed no significant differences.

#### 3.3.2 Dependence of bacterial count on the other factors.

An ordinary linear regression model was fitted with bacterial count as the response variable with the explanatory factors being the type of cropping system, electrical conductance, organic carbon, available nitrogen, and microbial biomass carbon.

**Fig 6:**
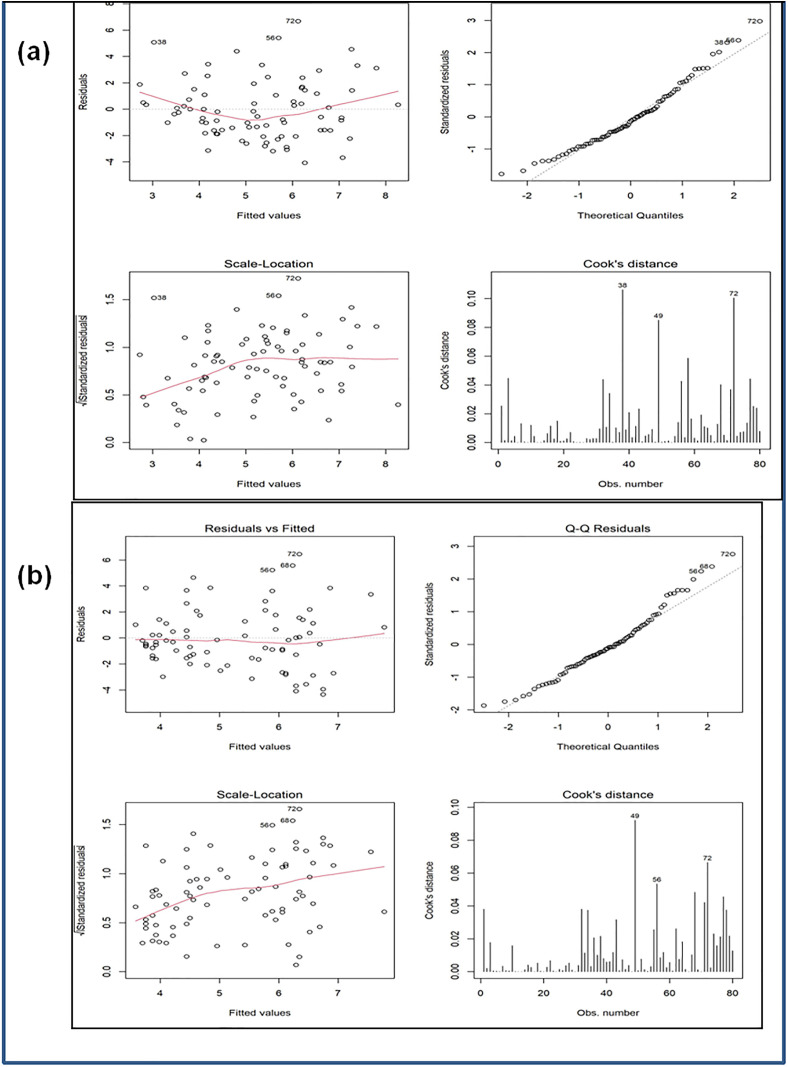
Residual Plots— (a) bac ct ~ type + elec cond + org car + avail nitro + micro biom car. (b) bac ct ~ type + avail nitro.

The results are given in Table 7 and the residual diagnostic plots are shown in Fig 6. The residual plots do not suggest any significant violation of the standard assumptions in linear regression. The p-value of the model is 0.0007163. At the 5% level, the only significant factors are the type of cropping system and available nitrogen. Fitting the linear model using only these two factors slightly improves the regression model as suggested by the residual plots. The AIC ((369.4779 and 370.1848 for the full and the reduced models respectively). The difference is insignificant to indicate any significant change in the model fit. Based on these observations, we can conclude that bacterial count depends significantly only on these two factors: type of cropping system and available nitrogen.

**Table 7:** Ordinary linear regression— bac ct ~ type + elec cond + org car + avail nitro + micro biom car.

	Estimate	Std. Error	z value	Pr(>|z|)
(Intercept)	−0.0412	1.4687	−0.03	0.9777
Type	1.8841	0.5326	3.54	0.0007
elec cond	0.1964	0.1511	1.30	0.1977
org car	0.6198	0.4487	1.38	0.1713
avail nitro	0.0747	0.0294	2.54	0.0131
micro biom car	0.0017	0.0009	1.90	0.0608

## 4 Discussions

In coastal areas around the world, agriculture is impacted by the long-term rise in salinity brought on by rising sea levels and increasing tropical storms [5]. This includes the Indian Sundarbans where salinization is the main factor for soil deterioration. Farmers use various agrarian methods to adjust to this. This change in land use and land cover causes alterations in the soil impacting on its microbiota. Due to restrictions on access to ground data, only secondary Landsat pictures have been utilized so far in spatial analysis to gain an understanding of the land use patterns and their effects on soil characteristics [21]. Most works have focused on the mangrove forests of the Indian Sundarbans. Very few studies address the agricultural aspects of the Indian Sundarbans, leading to a lack of reference for our research which centers specifically on its agricultural areas

Since the samples were collected from post-harvest soils, the pH was lower. Soil samples collected from post-harvested lands often tend to have lower range of pH due to organic acids released during mineralization and the cropping period and due to the effects of fertilizers like urea and DAP [22], which was evidently found in the analysis. Adding to this is the salinity levels predominant in these areas, as found during the study, influenced by the geographical factors like flooding during storms, and the type of irrigation methods used to manage salinity in agricultural fields [23,10,24]. All these studies add to demonstrate that salinity in agricultural land is primarily affected by irrigation practices, rainfall patterns, the frequency of saline water infusion, and the type of fertilizers used. Variations, if any, associated with agricultural practices, are generally minimal. Similarly, organic carbon and, consequently, organic matter levels varied geographically rather than being linked to specific agricultural systems.

Previously, studies had shown bacterial communities varying spatially due to salinity [25] which was also found in the study.In contrast to other studies, such as by Lucadamo et al., [26] and Lai et al., [27], which found enhanced microbial growth, microbial biomass carbon, and Available Nitrogen due to mixed cropping, our findings revealed that higher values for these factors were achieved in monocropping areas. This difference may be attributed to increased rainfall in the region and the practice of adding rice husks to the soil immediately after harvesting, which enhances field fertility alongside fertilizers, as noted by Marzouk et al., [28].

Li et al., [29] and Zhao et al., [30] illustrate how nitrogen affects bacterial growth which was seen in our study as well. Bacterial growth depends on Available Nitrogen, and high salinity frequently limits both. However, since the bacterial population in this soil consisted of salt-tolerant varieties (as found by the experiments), the overall thriving microbial population, especially bacteria had an enhancing effect on the Available Nitrogen of the agricultural soil.

The study shows that specific combinations of crops can improve soil quality, which in turn influences the microflora and microfauna. The study could be further expanded into understanding the difference in bacterial species population across the agricultural lands and how they are being shaped by its surroundings.

## 5 Conclusions

Due to the various land use systems in the Indian Sundarbans region, as well as susceptibility of the area to natural disasters and their consequences, it can also be deduced that the soil parameters in this region vary primarily geographically rather than due to agricultural systems. The intricacy of the soil systems and the susceptibilities of the Indian Sundarbans coastline, in addition to various farming methods, contribute to these exceptional outcomes. Further sampling could provide valuable insights into the specific causal factors involved. The salt-tolerant bacteria found in the soils of *Sitarampur, Sagar Islands, Patharpratima, and Bakkhali-Sibrampur* could be further analyzed for their potential benefits in salt mitigation. Since these isolated salt-tolerant bacterial varieties positively affect available nitrogen levels, their advantageous properties warrant additional study.

## Supporting information

S1 File It contains two tables S1 Table 1 and S1 Table 2. (DOCX)
