# Supplementary material for: The spatial distribution of salt tolerant bacteria and other soil parameters under different agricultural systems of the Indian Sundarbans
Source: PLoS One. 2025 Oct 8;20(10):e0333742. doi: 10.1371/journal.pone.0333742 (PMC12507285; doi:10.1371/journal.pone.0333742)
Supplement: S1 File — (DOCX) [file pone.0333742.s001.docx]

**Supplementary Information (S1)**

**S1 Table 1: Details of Sampling locations and types of fertilizers used in those areas:**

| **Sl No.** | **Nam of the Sampling area** | **Latitude of sampling locations** | **Longitude of sampling locations** | **Type of fertilizers used** |
| --- | --- | --- | --- | --- |
| 1 | Namkhana | 21° 43' 6.708" N | 88° 14' 37.248" E​ | Vermicompost, super potash; For vegetables: Urea, DAP, Borax spray |
|  |  | 21° 43' 15.564" N | 88° 14' 24.252" E​ |  |
| 2 | Kakdwip | 21° 54' 30.024" N | 88° 11' 3.2604" E​ | Khol fertilizer, potash, |
|  |  | 21° 54' 31.1508" N | 88° 11' 3.498" E​ |  |
| 3 | Patharpratima | 21° 44' 20.7816" N | 88° 20' 6.216" E​ | Shorshe khol, badam khol, cow dung, vermicompost, NPK |
|  |  | 21°44' 20.86296" N | 88°20'22.04088" E​ |  |
|  |  | 21° 44' 2.9076" N | 88° 20' 16.7244" E​ |  |
|  |  | 21°44' 13.59564" N | 88° 20' 18.3534" E​ |  |
| 4 | Sibrampur | 21°38' 38.75712" N | 88°15'25.26768" E​ | Urea, DAP, cow dung, khol |
|  |  | 21°38' 38.53248" N | 88°15'24.28632" E​ |  |
|  |  | 21°38' 36.69504" N | 88°15'23.99652" E​ |  |
| 5 | Debnibas near Bakkhali | 21°35' 54.18204" N | 88°16'40.52496" E​ | Cow dung, Diammonium Phosphate (DAP), Suphala,Urea, Potash |
|  |  | 21°36' 35.27604" N | 88°16'18.29568" E​ |  |
|  |  | 21° 36' 33.2064" N | 88° 16' 18.6528" E​ |  |
|  |  | 21° 36' 32.8356" N | 88° 16' 17.022" E​ |  |
| 6 | Kultoli | 21° 59' 55.6908" N | 88° 33' 59.0508" E​ | DAP, Urea, Mustard Khol, Manure |
|  |  | 21° 59' 48.2856" N | 88° 34' 6.744" E​ |  |
|  |  | 21° 59' 42.3816" N | 88° 34' 5.4912" E​ |  |
|  |  | 21° 59' 40.6464" N | 88° 34' 8.2596" E​ |  |
|  |  | 21° 59' 38.4612" N | 88° 34' 5.2716" E​ |  |
|  |  | 21° 59' 46.9428" N | 88° 33' 51.8076" E​ |  |
| 7 | Kirtankhali | 21° 42' 4.6152" N | 88° 6' 10.1988" E​ | Urea, khol, DAP |
|  |  | 21° 42' 5.5656" N | 88° 6' 10.7532" E​ |  |
|  |  | 21° 42' 6.0444" N | 88° 6' 10.1088" E​ |  |
|  |  | 21° 42' 4.41" N | 88° 6' 12.33" E​ |  |
|  |  | 21° 42' 2.6676" N | 88° 6' 12.0168" E​ |  |
|  |  | 21° 42' 2.5092" N | 88° 6' 12.1032" E​ |  |
|  |  | 21° 42' 2.3544" N | 88° 6' 12.0204" E​ |  |
|  |  | 21° 42' 2.214" N | 88° 6' 11.9916" E​ |  |
|  |  | 21° 42' 5.0652 " N | 88° 6' 9.2772" E​ |  |
|  |  | 21° 42' 6.7932" N | 88° 5' 41.1936" E​ |  |
|  |  | 21° 42' 7.1388" N | 88° 5' 40.4232" E​ |  |
| 8 | Kamalpur | 21° 42' 19.1304" N | 88° 6' 39.3084" E | Urea, DAP, Potash |
|  |  | 21° 42' 19.9368" N | 88° 6' 40.3164" E |  |
|  |  | 21° 42' 20.502" N | 88° 6' 41.4936" E |  |
| 9 | Chemaguri | 21° 40' 55.0452" N | 88° 7' 13.296" E | Urea, DAP, Potash |
|  |  | 21° 40' 55.4088" N | 88° 7' 12.1196" E |  |
| 10 | Purushottampur | 21° 40' 55.7904" N | 88° 7' 12.198" E | Urea, Potash |
|  |  | 21° 40' 57.1872" N | 88° 7' 14.628" E |  |
| 11 | South Haradhanpur | 21° 44' 2.328" N | 88° 7' 56.5536" E | Super Potash, Urea, Homemade from waste sometimes |
|  |  | 21° 44' 2.7924" N | 88° 7' 57.2268" E |  |
|  |  | 21° 44' 3.39" N | 88° 7' 57.4644" E |  |
| 12 | North Haradhanpur | 21° 45' 49.914" N | 88° 8' 18.0276" E | DAP, urea, Super potash, gomai, Suphala |
|  |  | 21° 45' 48.8592" N | 88° 8' 19.788" E |  |
|  |  | 21° 45' 49.5792" N | 88° 8' 10.114" E |  |
|  |  | 21° 45' 51.8472" N | 88° 8' 11.5764" E |  |
|  |  | 21° 45' 51.6924" N | 88° 8' 11.9436" E |  |
|  |  | 21° 45' 51.6636" N | 88° 8' 12.3972" E |  |
|  |  | 21° 45' 51.1596" N | 88° 8' 11.9976" E |  |
|  |  | 21° 45' 51.3324" N | 88° 8' 12.3648" E |  |
|  |  | 21° 45' 49.212" N | 88° 8' 19.0248" E |  |
| 13 | Digambari | 21° 44' 30.1632" N | 88° 4' 41.5308" E | Urea, 10 26, DAP; land tillage |
|  |  | 21° 44' 31.8624" N | 88° 4' 40.4148" E |  |
| 14 | Harinbari | 21° 44' 27.3444" N | 88° 5' 8.0268" E | Urea, Super phosphate, DAP |
|  |  | 21° 44' 26.2212" N | 88° 5' 2.9652" E |  |
|  |  | 21° 44' 24.216" N | 88° 4' 44.8392" E |  |
|  |  | 21° 44' 25.1088" N | 88° 4' 44.7744" E |  |
|  |  | 21° 44' 25.242" N | 88° 4' 43.662" E |  |
|  |  | 21° 44' 27.168" N | 88° 5' 11.3928" E |  |
| 15 | Sitarampur | 21° 38' 44.6172" N | 88° 24' 46.0296" E | Cow dung, Urea |
|  |  | 21° 38' 44.25" N | 88° 24' 45.324" E |  |
|  |  | 21° 38' 43.6956" N | 88° 24' 45.5472" E |  |
|  |  | 21° 38' 41.226" N | 88° 24' 32.3928" E |  |
|  |  | 21° 38' 42.5616" N | 88° 24' 30.438" E |  |
|  |  | 21° 38' 44.6208" N | 88° 24' 48.78" E |  |
|  |  | 21° 38' 47.3712" N | 88° 24' 46.8792" E |  |
| 16 | Buroburir Tot | 21° 37' 26.4936" N | 88° 23' 56.7096" E | Urea, Phosphate, DAP |
|  |  | 21° 37' 25.8384" N | 88° 23' 56.3712" E |  |
|  |  | 21° 37' 25.9608" N | 88° 23' 55.7088" E |  |
| 17 | Govardhanpur | 21° 36' 53.8308" N | 88° 23' 51.918" E | Urea, Dumur, DAP |
|  |  | 21° 36' 53.5608" N | 88° 23' 52.7424" E |  |
|  |  | 21° 36' 50.6704" N | 88° 23' 51.8972" E |  |
| 18 | G-Plot | 21° 40' 41.226" N | 88° 23' 49.3928" E | Gomai, Super phosphate, potash, sufala, DAP |
|  |  | 21° 40' 40.5616" N | 88° 23' 49.438" E |  |
|  |  | 21° 40' 39.8092" N | 88° 23' 48.228" E |  |
| 19 | Tot market | 21° 41' 57.436" N | 88° 24' 9.102" E | Superphosphate, sulphate, urea, Piri |
|  |  | 21° 41' 52.5444" N | 88° 23' 59.0856" E |  |
|  |  | 21° 41' 47.8788" N | 88° 23' 54.2184" E |  |
|  |  | 21° 41' 49.7904" N | 88° 24' 9.792" E |  |
|  |  | 21° 41' 58.0272" N | 88° 24' 13.7916" E |  |

**S1 Table 2: The raw values of the datasets given in Table 1.**

| SL. NO.​ | pH | | | EC | | | Bacterial count (10^6) | | | 20% NaCl tolerating bacteria | | | 10% NaCl tolerating bacteria | | | organic carbon | | | Available Nitrogen | | | Microbial Biomass C | | |
| --- | --- | --- | --- | --- | --- | --- | --- | --- | --- | --- | --- | --- | --- | --- | --- | --- | --- | --- | --- | --- | --- | --- | --- | --- |
| S1 | 5.4 | 5.4 | 5.42 | 5.29 | 5.24 | 5.4 | 7.1 | 8.02 | 7.68 | 3 | 3 | 3 | 11 | 12 | 7 | 1.82 | 1.85 | 1.78 | 10.49 | 10.32 | 10.54 | 780.15 | 781.12 | 782.18 |
| S2 | 6.62 | 6.67 | 6.63 | 4.07 | 4 | 4.08 | 5.15 | 5.25 | 5.2 | 3 | 4 | 2 | 10 | 12 | 11 | 2.08 | 2.11 | 2.08 | 16.74 | 16.86 | 16.61 | 498.86 | 495.03 | 484.69 |
| S3 | 7.2 | 7.21 | 7.37 | 4.08 | 4.04 | 4.15 | 2.94 | 3.06 | 3 | 1 | 1 | 1 | 6 | 6 | 3 | 2.94 | 2.96 | 2.9 | 35.54 | 35.61 | 35.47 | 390.76 | 386.54 | 394.98 |
| S4 | 6.87 | 6.79 | 6.96 | 6.25 | 6.34 | 6.19 | 3.22 | 3.38 | 3.3 | 1 | 1 | 1 | 6 | 6 | 6 | 0.94 | 0.99 | 0.89 | 10.45 | 10.51 | 10.39 | 150.52 | 156.48 | 144.56 |
| S5 | 7.13 | 7.12 | 7.14 | 4.86 | 4.78 | 4.94 | 6.03 | 5.97 | 6 | 1 | 2 | 0 | 2 | 3 | 1 | 1.27 | 1.35 | 1.19 | 54.39 | 54.25 | 54.5 | 510.88 | 515.72 | 506.04 |
| S6 | 7.85 | 7.74 | 7.93 | 4.17 | 4.24 | 4.1 | 3.51 | 3.69 | 3.6 | 0 | 0 | 0 | 1 | 2 | 0 | 1.51 | 1.55 | 1.47 | 13.59 | 13.69 | 13.49 | 480.85 | 474.91 | 486.79 |
| S7 | 7.2 | 7.26 | 7.14 | 5.03 | 4.96 | 5.1 | 4.64 | 4.56 | 4.6 | 1 | 2 | 0 | 5 | 2 | 2 | 1.08 | 1.11 | 1.05 | 7.32 | 7.26 | 7.38 | 340.91 | 343.18 | 338.64 |
| S8 | 7.47 | 7.46 | 7.48 | 5.55 | 5.58 | 5.52 | 3.18 | 3.22 | 3.2 | 2 | 2 | 2 | 5 | 6 | 4 | 1.02 | 1.06 | 0.98 | 10.45 | 10.52 | 10.38 | 241.21 | 243.09 | 239.33 |
| S9 | 7.11 | 7.13 | 7.09 | 3.8 | 3.86 | 3.8 | 4.98 | 5.02 | 5 | 1 | 1 | 1 | 3 | 4 | 2 | 2.68 | 2.65 | 2.62 | 23 | 23.15 | 22.85 | 657.03 | 656.54 | 669.52 |
| S10 | 6.97 | 6.95 | 7.05 | 4.49 | 4.42 | 4.56 | 1.02 | 1.1 | 1.06 | 2 | 1 | 3 | 4 | 4 | 4 | 1.95 | 1.91 | 1.87 | 15.67 | 15.74 | 15.63 | 600.97 | 605.32 | 596.62 |
| S11 | 5.39 | 5.36 | 5.42 | 2.25 | 2.3 | 2.2 | 3.9 | 3.88 | 3.92 | 0 | 0 | 0 | 1 | 1 | 1 | 2.76 | 2.73 | 2.7 | 18.82 | 18.91 | 18.73 | 1051.42 | 1048.21 | 1054.63 |
| S12 | 6.64 | 6.68 | 6.6 | 3.52 | 3.45 | 3.59 | 4.1 | 4.06 | 4.14 | 2 | 2 | 2 | 0 | 0 | 0 | 1.91 | 1.89 | 1.87 | 12.54 | 12.46 | 12.62 | 811.18 | 820.45 | 801.91 |
| S13 | 7 | 7.06 | 6.94 | 4.21 | 4.18 | 4.24 | 3.9 | 3.95 | 3.85 | 1 | 1 | 1 | 0 | 0 | 0 | 1.31 | 1.28 | 1.25 | 16.73 | 16.63 | 16.83 | 510.88 | 503.66 | 518.1 |
| S14 | 7.27 | 7.25 | 7.29 | 2.35 | 2.33 | 2.43 | 3.1 | 3.15 | 3.05 | 0 | 0 | 0 | 2 | 3 | 1 | 2.48 | 2.43 | 2.38 | 12.54 | 12.63 | 12.45 | 721.09 | 718.8 | 723.38 |
| S15 | 7.13 | 7.05 | 7.12 | 2.73 | 2.68 | 2.78 | 2.5 | 2.49 | 2.51 | 0 | 0 | 0 | 1 | 2 | 0 | 2.6 | 2.57 | 2.54 | 12.54 | 12.47 | 12.61 | 807.18 | 800.25 | 826.11 |
| S16 | 6.98 | 7.02 | 6.94 | 2.85 | 2.9 | 2.8 | 3.69 | 3.69 | 3.72 | 0 | 0 | 0 | 4 | 3 | 5 | 2.72 | 2.69 | 2.66 | 29.27 | 29.35 | 29.19 | 841.21 | 849.37 | 833.05 |
| S17 | 7.44 | 7.46 | 7.39 | 3.95 | 3.89 | 4.01 | 4.79 | 4.8 | 4.81 | 2 | 3 | 1 | 5 | 4 | 6 | 2.11 | 2.06 | 2.01 | 32.41 | 32.49 | 32.33 | 841.21 | 833.08 | 849.34 |
| S18 | 7 | 6.87 | 7.13 | 3.84 | 3.9 | 3.78 | 2.34 | 2.3 | 2.26 | 1 | 2 | 0 | 4 | 5 | 3 | 0.89 | 0.87 | 0.85 | 12.54 | 12.43 | 12.65 | 1171.54 | 1165.12 | 1177.96 |
| S19 | 7.29 | 7.32 | 7.23 | 4.18 | 4.1 | 4.23 | 4.68 | 4.7 | 4.72 | 0 | 0 | 0 | 6 | 7 | 5 | 1.6 | 1.66 | 1.72 | 18.82 | 18.75 | 18.89 | 1231.6 | 1225.49 | 1237.71 |
| S20 | 7.46 | 7.48 | 7.44 | 4.21 | 4.27 | 4.15 | 4.51 | 4.5 | 4.49 | 1 | 1 | 1 | 1 | 2 | 0 | 0.82 | 0.83 | 0.84 | 23 | 23.05 | 22.95 | 460.83 | 455.17 | 466.49 |
| S21 | 7.39 | 7.35 | 7.43 | 4.47 | 4.43 | 4.51 | 3.33 | 3.3 | 3.27 | 1 | 1 | 1 | 3 | 3 | 3 | 1.65 | 1.62 | 1.59 | 25.09 | 25.01 | 25.17 | 600.97 | 609.34 | 592.6 |
| S22 | 6.2 | 6.14 | 6.26 | 4.01 | 3.95 | 4.07 | 2.46 | 2.5 | 2.54 | 0 | 0 | 0 | 3 | 4 | 2 | 2.12 | 2.07 | 2.02 | 24.04 | 24.12 | 23.96 | 661.03 | 658.21 | 663.85 |
| S23 | 5.73 | 5.82 | 5.64 | 2.65 | 2.71 | 2.59 | 3.41 | 3.4 | 3.39 | 2 | 3 | 1 | 4 | 5 | 3 | 2.3 | 2.32 | 2.34 | 13.59 | 13.49 | 13.69 | 691.06 | 693.29 | 688.83 |
| S24 | 6.88 | 6.82 | 6.94 | 0.59 | 0.53 | 0.65 | 4.21 | 4.2 | 4.19 | 0 | 0 | 0 | 2 | 3 | 1 | 2.2 | 2.24 | 2.28 | 14.63 | 14.57 | 14.69 | 1111.48 | 1105.72 | 1117.24 |
| S25 | 7.38 | 7.42 | 7.34 | 0.45 | 0.4 | 0.5 | 3.32 | 3.3 | 3.28 | 0 | 0 | 0 | 1 | 1 | 1 | 1.97 | 1.99 | 2.01 | 19.86 | 19.79 | 19.93 | 480.85 | 472.19 | 489.51 |
| S26 | 7.23 | 7.25 | 7.17 | 1.52 | 1.58 | 1.46 | 3.82 | 3.8 | 3.78 | 1 | 1 | 1 | 2 | 1 | 3 | 1.32 | 1.35 | 1.38 | 24.04 | 24.09 | 23.99 | 552.92 | 560.48 | 545.36 |
| S27 | 7.24 | 7.36 | 7.12 | 2.53 | 2.46 | 2.6 | 5.37 | 5.4 | 5.43 | 0 | 0 | 0 | 1 | 1 | 1 | 2.18 | 2.18 | 2.18 | 14.63 | 14.54 | 14.72 | 595.11 | 600.97 | 606.83 |
| S28 | 7 | 6.92 | 7.08 | 1.8 | 1.85 | 1.75 | 2.31 | 2.3 | 2.29 | 2 | 1 | 3 | 3 | 3 | 3 | 2.11 | 2.05 | 2.17 | 13.59 | 13.67 | 13.51 | 421.88 | 416.79 | 411.7 |
| S29 | 6.42 | 6.29 | 6.55 | 0.87 | 0.92 | 0.82 | 3.1 | 3.15 | 3.05 | 1 | 1 | 1 | 0 | 0 | 0 | 2.49 | 2.46 | 2.52 | 10.45 | 10.38 | 10.52 | 1015.23 | 1021.39 | 1027.55 |
| S30 | 6.27 | 8.05 | 4.49 | 4.26 | 4.19 | 4.33 | 3.5 | 3.53 | 3.47 | 0 | 0 | 0 | 5 | 6 | 4 | 1.43 | 1.45 | 1.41 | 9.41 | 9.48 | 9.34 | 1194.37 | 1201.57 | 1208.77 |
| S31 | 6.65 | 6.11 | 7.19 | 0.54 | 0.48 | 0.6 | 2.9 | 2.88 | 2.92 | 2 | 2 | 2 | 6 | 8 | 4 | 1.08 | 1.09 | 1.07 | 23 | 22.95 | 23.05 | 1238.12 | 1231.6 | 1225.08 |
| S32 | 6.42 | 7.78 | 5.06 | 1.37 | 1.33 | 1.41 | 9.2 | 9.25 | 9.15 | 0 | 0 | 0 | 6 | 7 | 5 | 1.4 | 1.43 | 1.37 | 25.09 | 25.01 | 25.17 | 1120.78 | 1111.48 | 1102.18 |
| S33 | 7 | 7.5 | 6.5 | 0.51 | 0.56 | 0.46 | 7.1 | 7.14 | 7.06 | 0 | 0 | 0 | 4 | 5 | 3 | 2.67 | 2.63 | 2.71 | 23 | 22.92 | 23.08 | 1046.59 | 1051.42 | 1056.25 |
| S34 | 5.75 | 7.03 | 4.47 | 0.25 | 0.3 | 0.2 | 8.7 | 8.72 | 8.68 | 0 | 0 | 0 | 3 | 4 | 2 | 1.91 | 1.94 | 1.88 | 30.31 | 30.25 | 30.37 | 1135.47 | 1141.51 | 1147.55 |
| S35 | 7.47 | 5.89 | 9.05 | 2.34 | 2.37 | 2.31 | 3.1 | 3.05 | 3.15 | 1 | 1 | 1 | 4 | 5 | 3 | 1.46 | 1.48 | 1.44 | 24.04 | 23.93 | 24.15 | 208.56 | 210.58 | 212.6 |
| S36 | 6.68 | 7.12 | 6.24 | 1.24 | 1.17 | 1.31 | 2.5 | 2.56 | 2.44 | 0 | 0 | 0 | 1 | 1 | 1 | 1.92 | 1.88 | 1.96 | 33.45 | 33.51 | 33.39 | 303.12 | 300.67 | 298.22 |
| S37 | 6.54 | 6.45 | 6.63 | 1.24 | 1.29 | 1.19 | 2.7 | 2.64 | 2.76 | 0 | 0 | 0 | 2 | 2 | 2 | 2.23 | 2.25 | 2.21 | 29.27 | 29.38 | 29.16 | 327.45 | 330.7 | 333.95 |
| S38 | 6.88 | 6.58 | 7.18 | 1.99 | 2.05 | 1.93 | 8.1 | 8.05 | 8.15 | 0 | 0 | 0 | 3 | 4 | 2 | 0.43 | 0.47 | 0.39 | 23 | 22.88 | 23.12 | 431.62 | 420.79 | 409.96 |
| S39 | 7.09 | 6.97 | 7.21 | 1.53 | 1.47 | 1.59 | 6.7 | 6.71 | 6.69 | 0 | 0 | 0 | 3 | 2 | 4 | 1.81 | 1.79 | 1.83 | 26.13 | 26.18 | 26.08 | 501.74 | 510.88 | 520.02 |
| S40 | 5.92 | 7.18 | 4.66 | 0.3 | 0.25 | 0.35 | 6.4 | 6.38 | 6.42 | 0 | 0 | 0 | 1 | 1 | 1 | 1.69 | 1.68 | 1.7 | 27.18 | 27.08 | 27.28 | 366.9 | 360.73 | 354.56 |
| S41 | 7.98 | 5.85 | 10.11 | 3.85 | 3.92 | 3.78 | 6.7 | 6.74 | 6.66 | 1 | 1 | 1 | 8 | 8 | 8 | 2.45 | 2.46 | 2.44 | 7.32 | 7.29 | 7.35 | 582.11 | 590.76 | 599.41 |
| S42 | 6.18 | 7.88 | 4.48 | 4.08 | 4.02 | 4.14 | 3.4 | 3.46 | 3.34 | 0 | 0 | 0 | 10 | 9 | 11 | 1.87 | 1.85 | 1.89 | 19.86 | 19.9 | 19.82 | 243.98 | 240.61 | 237.24 |
| S43 | 7.92 | 6.3 | 9.54 | 3.11 | 3.16 | 3.06 | 2.4 | 2.37 | 2.43 | 2 | 2 | 2 | 12 | 14 | 10 | 2.14 | 2.18 | 2.1 | 9.41 | 9.33 | 9.49 | 330.57 | 320.49 | 310.41 |
| S44 | 7.53 | 7.94 | 7.12 | 3.21 | 3.26 | 3.16 | 6.6 | 6.61 | 6.59 | 0 | 0 | 0 | 10 | 15 | 5 | 2.27 | 2.29 | 2.25 | 16.73 | 16.82 | 16.64 | 548.13 | 540.91 | 533.69 |
| S45 | 6.12 | 7.49 | 4.75 | 4.08 | 4.13 | 4.03 | 4 | 4.05 | 3.95 | 1 | 1 | 1 | 3 | 4 | 2 | 2.15 | 2.14 | 2.16 | 11.5 | 11.45 | 11.55 | 119.16 | 120.49 | 121.82 |
| S46 | 5.7 | 6.05 | 5.35 | 3.14 | 3.09 | 3.19 | 5 | 4.97 | 5.03 | 0 | 0 | 0 | 3 | 3 | 3 | 2.55 | 2.52 | 2.58 | 15.68 | 15.57 | 15.79 | 842.75 | 841.21 | 839.67 |
| S47 | 5.22 | 5.63 | 4.81 | 6.19 | 6.13 | 6.25 | 7.9 | 7.85 | 7.95 | 1 | 2 | 0 | 9 | 8 | 10 | 1.7 | 1.68 | 1.72 | 24.04 | 24.12 | 23.96 | 180.55 | 185.2 | 175.9 |
| S48 | 7.3 | 5.3 | 9.3 | 7.46 | 7.39 | 7.53 | 6.3 | 6.28 | 6.32 | 2 | 2 | 2 | 10 | 11 | 9 | 0.98 | 1.02 | 0.94 | 23 | 23.05 | 22.95 | 240.61 | 237.42 | 243.8 |
| S49 | 5.47 | 7.38 | 3.56 | 6.75 | 6.82 | 6.68 | 10.9 | 10.92 | 10.88 | 1 | 1 | 1 | 13 | 11 | 15 | 1.44 | 1.49 | 1.39 | 45.99 | 46.05 | 45.93 | 180.55 | 177.19 | 183.91 |
| S50 | 5.67 | 5.55 | 5.79 | 5.44 | 5.38 | 5.5 | 6.6 | 6.56 | 6.64 | 2 | 3 | 1 | 7 | 8 | 6 | 1.75 | 1.8 | 1.7 | 16.73 | 16.62 | 16.84 | 570.94 | 579.45 | 562.43 |
| S51 | 5.32 | 5.61 | 5.03 | 4.23 | 4.29 | 4.17 | 8.6 | 8.57 | 8.63 | 4 | 4 | 4 | 7 | 7 | 7 | 1.69 | 1.68 | 1.7 | 50.18 | 50.11 | 50.25 | 480.85 | 472.8 | 488.9 |
| S52 | 7.6 | 5.27 | 9.93 | 4.65 | 4.71 | 4.59 | 5 | 5.01 | 4.99 | 1 | 1 | 1 | 0 | 0 | 0 | 1.18 | 1.2 | 1.16 | 13.69 | 13.49 | 13.59 | 781.15 | 775.11 | 787.19 |
| S53 | 7.41 | 7.51 | 7.31 | 4.83 | 4.78 | 4.88 | 6.9 | 6.94 | 6.86 | 2 | 2 | 2 | 4 | 4 | 4 | 0.99 | 1 | 0.98 | 27.25 | 27.11 | 27.18 | 811.18 | 807.28 | 815.08 |
| S54 | 7.79 | 7.43 | 7.61 | 4.75 | 4.68 | 4.82 | 7.8 | 7.76 | 7.84 | 0 | 0 | 0 | 2 | 3 | 1 | 1.05 | 1.08 | 1.02 | 25.17 | 25.01 | 25.09 | 540.91 | 545.53 | 536.29 |
| S55 | 7.32 | 7.68 | 7.5 | 3.95 | 4.01 | 3.89 | 9.5 | 9.46 | 9.54 | 0 | 0 | 0 | 3 | 2 | 4 | 1.76 | 1.83 | 1.69 | 15.63 | 15.73 | 15.68 | 1021.39 | 1013.98 | 1028.8 |
| S56 | 7.31 | 7.55 | 7.43 | 4.01 | 3.94 | 4.08 | 11.1 | 11.09 | 11.11 | 1 | 1 | 1 | 2 | 2 | 2 | 1.78 | 1.79 | 1.77 | 15.76 | 15.6 | 15.68 | 480.85 | 492.07 | 469.63 |
| S57 | 7.52 | 7.34 | 7.43 | 3.47 | 3.52 | 3.42 | 8.7 | 8.69 | 8.71 | 3 | 4 | 2 | 6 | 5 | 7 | 1.66 | 1.66 | 1.66 | 27.31 | 27.05 | 27.18 | 1021.39 | 1017.45 | 1025.33 |
| S58 | 7.59 | 7.41 | 7.5 | 5.76 | 5.7 | 5.82 | 3.4 | 3.31 | 3.49 | 1 | 1 | 1 | 5 | 7 | 3 | 1.22 | 1.24 | 1.2 | 18.74 | 18.9 | 18.82 | 1171.54 | 1162.8 | 1180.28 |
| S59 | 7.38 | 7.52 | 7.45 | 3.26 | 3.21 | 3.31 | 5 | 5.05 | 4.95 | 1 | 1 | 1 | 3 | 4 | 2 | 2.88 | 2.93 | 2.83 | 22.93 | 23.07 | 23 | 751.12 | 759.33 | 742.91 |
| S60 | 7.29 | 7.41 | 7.35 | 3.24 | 3.18 | 3.3 | 7.7 | 7.75 | 7.65 | 2 | 2 | 2 | 3 | 3 | 3 | 2.58 | 2.57 | 2.59 | 16.82 | 16.64 | 16.73 | 721.09 | 726.05 | 716.13 |
| S61 | 7.43 | 7.31 | 7.37 | 2.57 | 2.62 | 2.52 | 6.2 | 6.18 | 6.22 | 0 | 0 | 0 | 2 | 4 | 0 | 1.36 | 1.39 | 1.33 | 30.37 | 30.25 | 30.31 | 961.33 | 959.12 | 963.54 |
| S62 | 7.68 | 7.28 | 7.48 | 2.27 | 2.32 | 2.22 | 2.2 | 2.19 | 2.21 | 0 | 0 | 0 | 1 | 1 | 1 | 1.24 | 1.27 | 1.21 | 22.94 | 23.06 | 23 | 901.27 | 895.67 | 906.87 |
| S63 | 7.44 | 7.5 | 7.47 | 3.35 | 3.28 | 3.42 | 4 | 4.02 | 3.98 | 0 | 0 | 0 | 2 | 2 | 2 | 1.5 | 1.53 | 1.47 | 9.35 | 9.47 | 9.41 | 1171.54 | 1161.41 | 1181.67 |
| S64 | 6.99 | 7.45 | 7.22 | 2.8 | 2.85 | 2.75 | 8.6 | 8.64 | 8.56 | 0 | 0 | 0 | 3 | 5 | 1 | 1.56 | 1.6 | 1.52 | 13.56 | 13.62 | 13.59 | 1111.48 | 1103.22 | 1119.74 |
| S65 | 7.68 | 7.3 | 7.49 | 1.31 | 1.37 | 1.25 | 5.1 | 5.06 | 5.14 | 0 | 0 | 0 | 4 | 4 | 4 | 2.32 | 2.29 | 2.35 | 18.73 | 18.91 | 18.82 | 1051.42 | 1058.33 | 1044.51 |
| S66 | 5.84 | 7.48 | 6.66 | 0.53 | 0.46 | 0.6 | 5.6 | 5.63 | 5.57 | 0 | 0 | 0 | 5 | 5 | 5 | 1.45 | 1.52 | 1.38 | 7.39 | 7.25 | 7.32 | 1081.45 | 1090.59 | 1072.31 |
| S67 | 8.08 | 6.54 | 7.31 | 0.52 | 0.48 | 0.56 | 7.9 | 7.92 | 7.88 | 2 | 2 | 2 | 4 | 6 | 2 | 1.57 | 1.63 | 1.51 | 13.69 | 13.49 | 13.59 | 931.3 | 936.44 | 926.16 |
| S68 | 7.07 | 7.23 | 7.15 | 3.19 | 3.25 | 3.13 | 11.8 | 11.78 | 11.82 | 2 | 3 | 1 | 4 | 5 | 3 | 2.12 | 2.13 | 2.11 | 22 | 21.9 | 21.95 | 1111.48 | 1100.16 | 1122.8 |
| S69 | 6.56 | 7.14 | 6.85 | 3.41 | 3.36 | 3.46 | 5.2 | 5.18 | 5.22 | 1 | 1 | 1 | 7 | 7 | 7 | 1.54 | 1.55 | 1.53 | 18.89 | 18.75 | 18.82 | 1171.54 | 1158.21 | 1184.87 |
| S70 | 8 | 6.72 | 7.36 | 0.92 | 0.87 | 0.97 | 6.4 | 6.41 | 6.39 | 2 | 2 | 2 | 13 | 16 | 10 | 1.45 | 1.47 | 1.43 | 24.07 | 24.01 | 24.04 | 1411.78 | 1415.32 | 1408.24 |
| S71 | 7.62 | 7.26 | 7.44 | 0.21 | 0.26 | 0.16 | 10.7 | 10.68 | 10.72 | 3 | 4 | 2 | 7 | 6 | 8 | 1.65 | 1.67 | 1.63 | 33.4 | 33.5 | 33.45 | 1201.57 | 1190.47 | 1212.67 |
| S72 | 7.58 | 7.56 | 7.6 | 0.27 | 0.2 | 0.31 | 12.8 | 12.75 | 12.85 | 0 | 0 | 0 | 6 | 8 | 4 | 2.08 | 2.06 | 2.1 | 24.1 | 23.98 | 24.04 | 691.06 | 695.32 | 686.8 |
| S73 | 7.53 | 7.59 | 7.47 | 0.3 | 0.35 | 0.25 | 7.7 | 7.74 | 7.66 | 0 | 0 | 0 | 9 | 8 | 10 | 1.87 | 1.83 | 1.91 | 28.29 | 28.15 | 28.22 | 661.03 | 657.8 | 664.26 |
| S74 | 7.98 | 7.48 | 8.48 | 0.5 | 0.45 | 0.55 | 4.2 | 4.24 | 4.16 | 0 | 0 | 0 | 3 | 4 | 2 | 0.68 | 0.69 | 0.67 | 34.5 | 34.45 | 34.55 | 300.67 | 302.99 | 298.35 |
| S75 | 7.93 | 7.95 | 7.91 | 0.37 | 0.42 | 0.32 | 3.7 | 3.72 | 3.68 | 0 | 0 | 0 | 1 | 0 | 2 | 0.72 | 0.74 | 0.7 | 28.22 | 28.27 | 28.17 | 360.73 | 367.25 | 354.21 |
| S76 | 7.81 | 7.9 | 7.72 | 2.56 | 2.51 | 2.61 | 2.6 | 2.62 | 2.58 | 0 | 0 | 0 | 5 | 4 | 6 | 0.89 | 0.92 | 0.86 | 23 | 23.07 | 22.93 | 480.85 | 478.13 | 483.57 |
| S77 | 8.19 | 8.12 | 8.26 | 0.2 | 0.26 | 0.14 | 2.4 | 2.35 | 2.45 | 0 | 0 | 0 | 5 | 6 | 4 | 0.4 | 0.42 | 0.38 | 31.36 | 31.44 | 31.28 | 670.64 | 675.84 | 665.44 |
| S78 | 5.47 | 5.47 | 5.47 | 0.23 | 0.28 | 0.18 | 2.8 | 2.75 | 2.85 | 0 | 0 | 0 | 4 | 5 | 3 | 1.14 | 1.18 | 1.1 | 31.36 | 31.28 | 31.44 | 570.94 | 567.24 | 574.64 |
| S79 | 5.52 | 5.59 | 5.45 | 0.17 | 0.22 | 0.12 | 2.9 | 2.92 | 2.88 | 0 | 0 | 0 | 4 | 5 | 3 | 0.52 | 0.55 | 0.49 | 26.13 | 26.08 | 26.18 | 780.55 | 785.41 | 775.69 |
| S80 | 4.98 | 4.87 | 5.09 | 0.8 | 0.86 | 0.76 | 3.3 | 3.28 | 3.32 | 0 | 0 | 0 | 7 | 8 | 6 | 1.28 | 1.31 | 1.25 | 19.86 | 19.94 | 19.78 | 661.03 | 661.9 | 660.16 |
